# A perspective-based analysis of attachment from prenatal period to second year postnatal life

**DOI:** 10.3389/fpsyg.2024.1296242

**Published:** 2024-05-22

**Authors:** Erica Santaguida, Massimo Bergamasco

**Affiliations:** Institute of Mechanical Intelligence, Scuola Superiore Sant’Anna, Pisa, Italy

**Keywords:** attachment development, prenatal attachment, genetics, neurobiology, cognition

## Abstract

Attachment is one of the foundational themes in the history of the psychological development of human beings. For this reason, we assume that it must be approached by taking into account multiple scientific perspectives. The present review aims at analyzing the state of the art regarding the genetic, neurobiological and cognitive mechanisms underlying the development of attachment bonding, considering the child as the frame of reference. We hypothesize that attachment may be present in prototypical forms even in the prenatal period, thus our analysis has a temporal origin in the intrauterine period preceding birth. The intrauterine period is assumed to be a period of maximum sensitivity to stimuli and in particular to those coming from a potential primary caregiver: the biological mother. We conclude with a reframing of the state of the art and propose that future research work would benefit from a superordinate model of attachment, capable of containing and regulating all its components and variables.

## 1 Introduction

Attachment theory (Bowlby, 1969/1982) is a unique example in the psychoanalytic landscape, as it represents a point of connection between general psychology and clinical psychodynamic theory (Fonagy and Target, 2003). While the historical approach to understanding psychic development often involved retrospective analysis, focusing on the dynamics of adults (e.g., M. Klein, A. Freud), John Bowlby took a different approach (Bowlby, 1944) and directed his attention towards the study of child development by means of direct observation. According to its theoretical formulation, attachment is a behavioral system (Bowlby, 1969/1982), therefore a system animated by an intrinsic motivation, and not reducible to other drives (e.g., sexuality or hunger), that is activated in case of disturbances and de-activated when the internal balance is restored. In the decades following Bowlby’s pioneering work, new experimental methodologies and further reflections have led to the overcoming and enrichment of some concepts of attachment theory, both in the clinical and research fields. Sroufe (Sroufe and Waters, 1977; Sroufe, 1996), among other authors, has provided insightful reinterpretations of the theory, noting that the attachment system aims to attain a sense of perceived security. This implies that internal cues of distress hold the same significance as external cues of physical separation from the caregiver in triggering the attachment system’s response.

**Figure 1:**
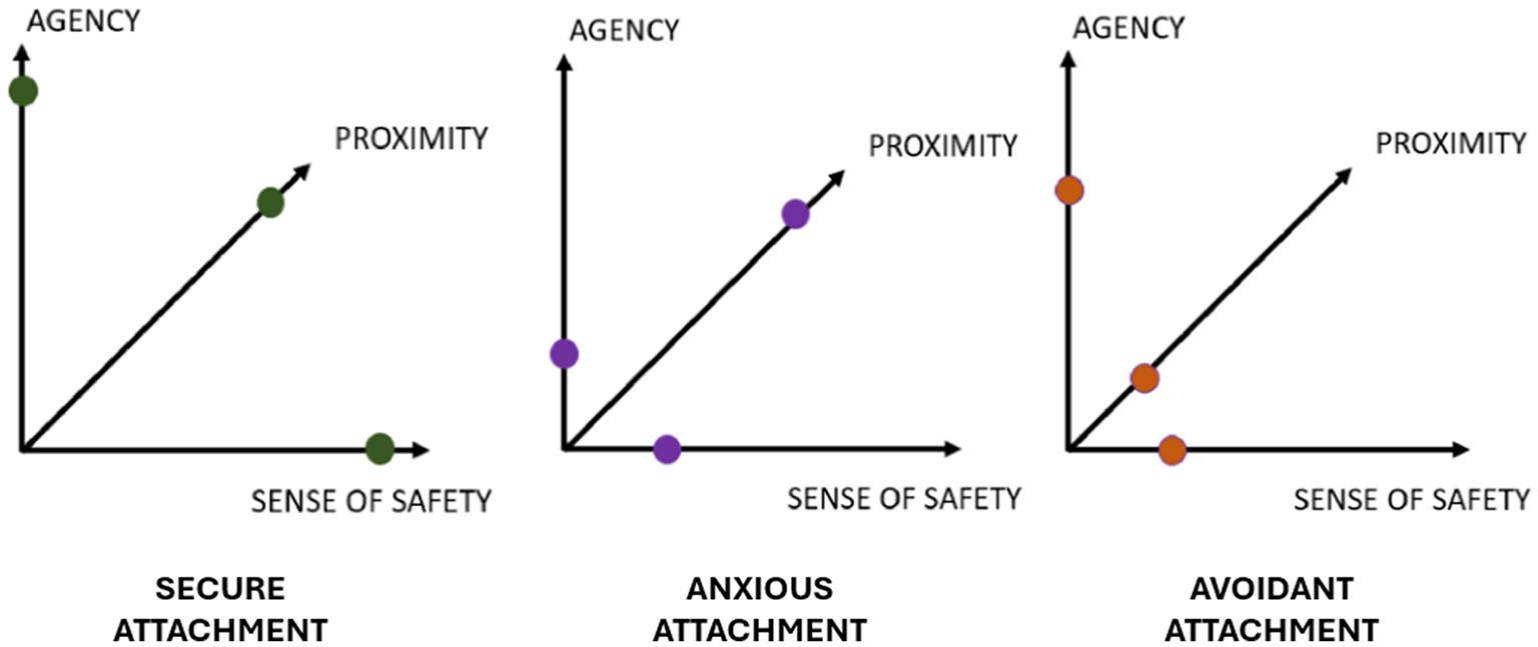
Attachment styles. Schematic representation of secure, insecure anxious, insecure avoidant attachment styles, according to three main dimensions: degree of perceived safety, degree of search for proximity to the caregiver, and sense of agency.

The caregiver’s ability to respond to the infant’s needs plays a crucial role in the formation of the attachment behavioral system. However, it is not the only determining factor in establishing an attachment style (Cassidy et al., 2013; Thompson, 2021). The latter is recognizable by the end of the second year and is conceptualized into two macro-categories: secure and insecure attachment, depending on whether repeated experiences of interaction with the primary caregiver have been characterized by parental sensitivity and availability or parental irresponsiveness and/or inconsistency, respectively (Ainsworth, 1967; Bowlby, 1969/1982; Ainsworth et al., 1978). Various classifications of attachment styles have emerged over the decades (e.g., Ainsworth et al., 1978; Main and Solomon, 1990; Crittenden and Landini, 2011) united by the consideration of the axes of anxiety and avoidance as key dimensions in its assessment. Different attachment styles correspond to distinct emotional regulation strategies and cognitive models that influence appraisal and prediction of environmental contingencies as well as the organization of behavioral policies (Cassidy and Kobak, 1988; Brennan et al., 1998; Bifulco and Thomas, 2012) (see Figure 1).

Internal Working Models (IWM) are a milestone of the attachment theory and they were conceptualized by Bowlby as internal representations of the world that can be cognitively manipulated to allow for the modification of the variables they represent. IWM contain, gather, and organize information with the aim of making predictions about how to achieve certain goals, including maintaining proximity to the caregiver in the context of attachment. The attachment system, in order to select the most suitable behavioral policy for the purpose, uses available IWM of the caregiver, of the Self, and of the world, which are based on early repeated interactive experience. IWM contribute to the perpetuation of strategies related to certain attachment styles even into adulthood (Coan, 2010; Sheinbaum et al., 2015). From a clinical point of view, there are many studies in the scientific literature that observed the modalities and the pervasiveness with which certain attachment styles influence the development of pathological traits (e.g., Mikulincer and Shaver, 2012; Schindler, 2019; Stauffer et al., 2020; Teng et al., 2020), with particular reference to the disorganized style (e.g., Main and Solomon, 1986; MacDonald et al., 2008; Granqvist et al., 2017; van Hoof et al., 2019; Decarli et al., 2022).

Attachment theory has rapidly evolved into an interdisciplinary field of study that attracts research focused on understanding the human being. Various disciplines have contributed to the exploration of the mechanisms of formation of the attachment system, often focusing on the quality of parental care and its impact on child development. The result is a vast and varied scientific literature that offers multiple perspectives on the evolutionary importance of the attachment system. While it’s undisputed that social environmental influences, such as the quality of care received, play a crucial role in defining attachment style, what are the internal neurobiological and psychological processes within the infant that cooperate in defining this system? As we will see in the subsequent sections of this work, studies from neuroscience, genetics, and cognitive psychology are some of the research fields that have contributed to clarifying how, through a complex interaction of factors, the infant organizes early experiences of the social world into a determined attachment system.

This multidisciplinary approach has led to an improved understanding of the internal mechanisms that determine attachment. For instance, studies on the neurobiology of attachment have highlighted how early experiences influence brain development and how this, in turn, impacts attachment patterns. Similarly, psychological research has explored how IWM formed through interactions with caregivers, guide a child’s expectations and reactions in situations of stress or separation. Despite the richness of the multidisciplinary literature, there remains the challenge of integrating knowledge from diverse perspectives into a single multidisciplinary model of attachment. Such an integrated model should consider both biological and psychological factors in attachment system formation and evolution to elucidate how internal processes interact and contribute to shaping the attachment system, providing a comprehensive understanding of this critical aspect of human development.

**Figure 2:**
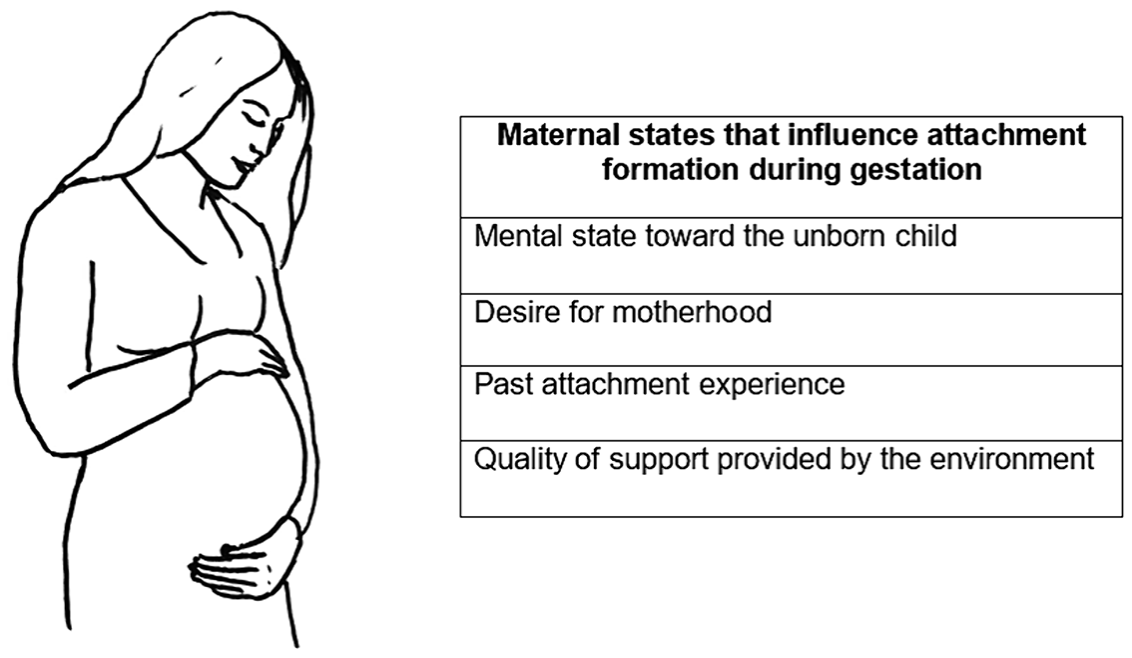
Maternal conditions that influence the attachment bond with the baby starting from the gestational period.

An integrated theoretical framework could contribute to another significant aspect of attachment research: its evolutionary origins. Here the question is: when do we start to build an attachment system? Bowlby (1969/1982) originally described attachment development as a process of progressive stages that take place during the first three years of life (Ainsworth et al., 1978). However, the study of attachment has more recently been extended to the prenatal period, and it is well accepted that factors including the mother’s mental state toward the unborn child, her desire for motherhood, her past attachment experience, and the quality of support provided by the environment, affect the development of postnatal attachment (Cranley, 1981; Zimerman and Doan, 2003; Smorti et al., 2020) (see Figure 2).

**Figure 3:**
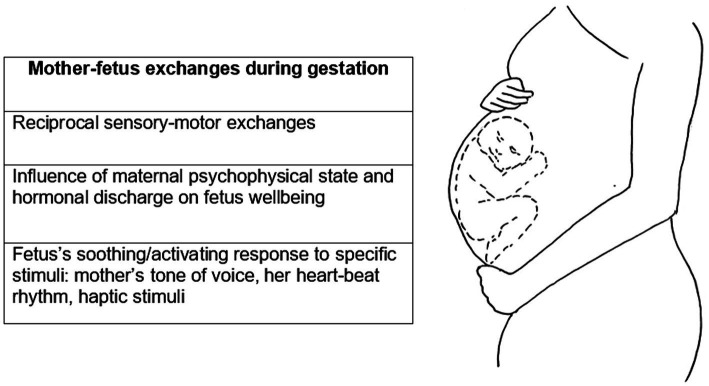
Mother-fetus exchanges and reciprocal influence.

Considering the child as a frame of reference, the classical conception of IWM is not explanatory in understanding whether and how the prenatal experience of cohabitation within the maternal body influences the development of the attachment system in the child. As will be further explored in the following paragraphs, during this period, there is already a form of communication between mother and child perpetrated through sensory-motor (Marx and Nagy, 2015, 2017) and chemical inputs (Sandman et al., 1997; DiPietro, 2012). The maternal psychophysical state directly and indirectly affects the well-being of the fetus through hormonal discharges [e.g., cortisol discharges triggered by stressful conditions (Kinsella and Monk, 2009; DiPietro, 2012; Zietlow et al., 2019)] and through sensory stimuli such as the mother’s tone of voice, her heartbeat rhythm, and haptic stimuli that come from her belly rubbing and that become reciprocal when fetal movements become externally perceptible in the fifth month. The fetus is far from passive and acts or responds to stimuli differentially, showing the ability of distinguishing between pleasant and unpleasant, soothing or activating tactile and auditory stimuli, and between stimuli from mother, other familiar individuals or strangers (Fifer and Moon, 1988; Marx and Nagy, 2015, 2017) (see Figure 3).

The fetus uses information gathered during intrauterine life to predict the safety of the external environment and to address specific caregiving figures after birth. This is evident from their retainment of familiar voices and a preference for the smell and voice of the mother (DeCasper and Fifer, 1980; Fifer and Moon, 1988; Sai, 2005; Vaglio, 2009; Streri et al., 2013).

The gestational period represents a time of extremely intense and unique interaction between the fetus and the mother, potentially the primary caregiver of the newborn. Despite various studies on the prenatal period highlighting the presence of complex neurobiological and cognitive functions in the fetus, the precise role of this period in the development of a specific attachment style remains somewhat unclear in the current literature (Lavelli, 2007).

In a new theoretical framework that entails the concept of IWM as embodied representations of the world and affective bonds deeply rooted in the body and somatic experience, could be more useful in understanding how the mother-fetus interaction lays the relational foundation for the extra-uterine mother-infant attachment bonding.

## 2 Objectives and hypotheses of the study

Attachment is a complex system, whose development concerns various levels of organization. In this work, we propose to adopt three ‘bottom-up’ perspectives—genetic, neuroscientific, and cognitive—to explore the mechanisms that cooperate during the development of the attachment system in children. This choice is based on the importance of these areas in understanding how innate predispositions, the biological bases of affective relationships and cognitive processes contribute to the formation, interpretation, and lasting influence of primary attachment relationships. In particular, they are useful for answering three questions: Does a child have an innate predisposition to develop certain attachment styles? How does the body, especially the nervous system, shape and get shaped by the attachment experience? Through which cognitive tools is the caregiving experience processed and represented? Previous works, such as those by Cassidy and Shaver (2008, 2018), have examined a wide range of fields of study, including those of our interest, to outline the state of the art of attachment research. However, our work does not aim to be exhaustive on all possible ‘bottom-up’ approaches but focuses on some key perspectives to offer a comprehensive and multidimensional view of the development of attachment from the earliest stages of life.

The first guiding hypotheses of our work is that the ‘bottom-up’ perspectives call for the need for a neuro-cognitive overarching model capable of integrating studies from different disciplines to explain the mechanisms of development of the attachment system in children. Such a model, operating in a top-down manner, could synthesize and regulate according to universally valid principles the various bottom-up perspectives. We then analyze studies from these three areas to determine whether it is possible to achieve convergence between these perspectives, suggesting the need for an integrated model, or whether a lack of overlap emerges, indicating difficulties or irrelevance in integration.

While exploring the bottom-up perspectives and the possibility of an integrated model of attachment, we aim at confronting another significant challenge: exploring the possibility of extending the temporal origin of an individual’s attachment system development to the prenatal period.

As anticipated, it is acknowledged that prenatal attachment between mother and child significantly influences the future mother–child relationship (Cranley, 1981; Zimerman and Doan, 2003; Smorti et al., 2020). This effect mainly stems from the mental representations that the mother develops based on her pregnancy experience, the family support she receives, and her past attachment experiences. Similarly, the quality of the interaction between the caregiver and child during the early years of life is a determining factor (even if it is not sufficient itself) in the development of a specific attachment style (Cassidy et al., 2013). However, existing literature offers few studies exploring the presence of a rudimentary attachment system in the fetus, related to the quality of the symbiotic relationship with the biological mother. In our review work, we analyze scientific research supporting our study’s second hypothesis: the possible existence of prenatal attachment system in the fetus. According to this hypothesis, the roots of developing security or insecurity in the attachment system are grounded in the prenatal period, a time when the highly sensitive and plastic fetus assimilates crucial information from the mother’s body that hosts it, from the own, and from the external environment filtered through the maternal uterus. From this information, the fetus might form pre-cognitive, biological representations of security and insecurity, laying the groundwork for an attachment style that mainly manifests in the first two years of the child’s life. In the following sections of the article, studies of the prenatal period based on the aforementioned perspectives will be examined to explore the possibility to consider the existence of pre-cognitive representations of security and insecurity, both regarding the world in general and in relation to the biological mother. Although it is known that the fetus and newborn probably do not possess the ability to distinguish between self and others (Assmann, 2020), our hypothesis does not necessarily rely on this distinctive capability. Instead, it focuses on the idea that the fetus can develop differentiated responses and predictions to specific stimuli, such as the mother’s voice, to be interpreted as precursors of attachment. These responses could lay the foundation for the subsequent development of attachment relationships, once the child develops greater self-awareness. Therefore, one of the goals of this study is to observe how the prenatal experience fits into a continuum with the postnatal experience in defining an attachment style within the first two years of life.

We have chosen to limit the analysis of papers to the second year of life. This choice is mainly based on the fact that this period represents a heightened sensitivity phase, where the infant’s experiences have a profound and lasting impact on their attachment style and overall psychological development.

In fact, *t* is typically during this period that a child’s attachment style becomes established (e.g., Haselbeck et al., 2018).

## 3 Methods

**Figure 4:**
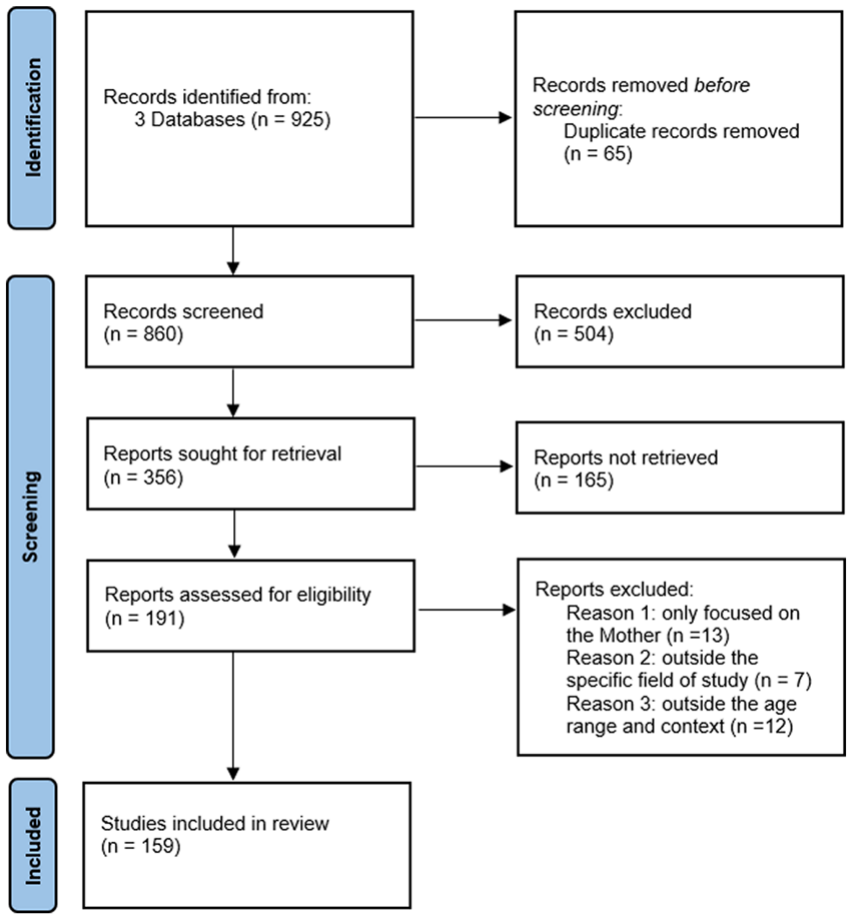
PRISMA flow diagram outlining the identification, screening, eligibility, and inclusion criteria for the studies found within the scope of this review.

For the present work we have included studies covering a wide temporal range, from Bowlby’s fundamental research in 1944 to studies published up to 2023. While the majority of the studies included are from the period 2003 to 2023, we have also included seminal and historical works. This approach has allowed us to analyze not only the latest discoveries but also to recognize and integrate the theoretical foundations and fundamental contributions provided by earlier studies in the field of human attachment. We conducted a narrative literature review following the guidelines of the Preferred Reporting Items for Systematic Reviews and Meta-Analyses (PRISMA) (Moher et al., 2015) (see Figure 4).

The keywords used for the search include: “genetics of attachment,” “mother-fetus interaction,” “neurobiology of attachment,” “prenatal attachment,” “prenatal cognition,” “prenatal sensitivity and responsivity,” “cognitive processes and attachment.” We utilized the databases PsychInfo, PubMed, and Google Scholar. The search process initially identified 857 papers. We applied specific inclusion and exclusion criteria. We included studies that explored neurobiological, genetic, and/or cognitive aspects involved in the development of the attachment bond in the first two years of life, as well as those investigating neurobiological, genetic, and cognitive aspects in the fetus and their interaction with stimuli from the uterus and the external world. On the other hand, we excluded studies focused on aspects of attachment not pertinent to the specific scope of our paper, such as those centered on attachment in adulthood, in specific clinical contexts, or studies that focused exclusively on the maternal system without including the child or fetus. After screening and assessment of suitability according to the established criteria, the number of references was reduced to 159, which represent the basis for our analysis and synthesis (Table 1).

**Table 1:** Inclusion criteria for analyzed articles.

	Infancy			Peri-natal					
Authors	GEN	NB	COG	GEN	NB	COG	Theoretical basis	Theoretical support to hypotheses	Support to methodology
Ainsworth (1967)							X		
Ainsworth et al. (1978)							X		
Assmann (2020)				X					
Atzil et al. (2018)		X							
Atzil et al. (2011)		X							
Bakermans-Kranenburg et al. (2012)	X								
Bakermans-Kranenburg et al. (2011)	X								
Bakermans-Kranenburg and Van IJzendoorn (2016)	X								
Barker (1990)				X					
Barker and Godfrey (2004)				X					
Barker and Osmond (1986)				X					
Barker et al. (1993)				X					
Barry et al. (2008)	X								
Beckes et al. (2015)			X						
Bifulco and Thomas (2012)							X		
Bokhorst et al. (2003)	X								
Bosmans et al. (2020)			X						
Bosmans et al. (2018)	X								
Bowlby (1944)							X		
Bowlby (1969/1982)							X		
Bowlby (1973)							X		
Bowlby (1980)							X		
Bowlby (1988)							X		
Brennan et al. (1998)							X		
Cassidy et al. (2013)							X		
Cassidy and Kobak (1988)							X		
Cassidy and Shaver (2008)	X	X	X						
Cassidy and Shaver (2018)	X	X	X						
Champagne (2010)								X	
Choi et al. (2018)		X							
Ciaunica et al. (2021)					X				
Coan (2010)							X		
Coan (2016)							X		
Coan and Sbarra (2015)		X							
Cole (2013)	X	X							
Conradt et al. (2018)				X	X				
Craig et al. (2021)	X								
Craik (1943)							X		
Cranley (1981)							X		
Crittenden and Landini (2011)							X		
Dahlen et al. (2013)				X					
Decarli et al. (2022)							X		
DeCasper and Fifer (1980)						X			
Delafield-Butt and Trevarthen (2013)						X			
DiPietro (2012)					X				
DiPietro et al. (2021)					X				
Donnellan et al. (2008)	X								
Ebner et al. (2019)	X								
Ehrlich et al. (2016)		X							
Ein-Dor et al. (2018)	X								
Eisenberger et al. (2011)		X							
Engel and Gunnar (2020)		X							
Epel et al. (2018)								X	
Erkoreka et al. (2021)	X								
Fearon et al. (2014)	X								
Fearon et al. (2017)	X	X							
Feldman (2017)		X							
Fifer and Moon (1988)						X			
Flom and Johnson (2011)						X			
Fonagy and Target (2003)							X		
Forray (2016)				X					
Franklin et al. (2012)				X					
Friston et al. (2006)								X	
Fujiwara et al. (2019)				X					
Gilissen et al. (2008)	X								
Granier-Deferre et al. (2011)						X			
Granqvist et al. (2017)							X		
Haselbeck et al. (2018)		X			X				
Haviland and Lelwica (1987)			X						
Hurlemann and Scheele (2016)	X								
Hygen et al. (2014)	X								
Jarvis (2022)		X							
Jiang et al. (2019)	X								
Jinxia and Xuxia (2022)		X							
Johnson et al. (2018)		X							
Kadic and Kurjak (2018)						X			
Kinsella and Monk (2009)					X				
Kochanska et al. (2009)	X								
Lagercrantz (2007)						X			
Lamb and Malkin (1986)			X						
Lavelli (2007)						X			
Lecompte et al. (2021)	X								
Linderkamp and Linderkamp-Skoruppa (2021)				X					
Linnér et al. (2020)	X								
Luijk et al. (2011)	X								
MacDonald et al. (2008)							X		
Main and Solomon (1986)							X		
Main and Solomon (1990)							X		
Marx and Nagy (2015)						X			
Marx and Nagy (2017)						X			
Meaney and Szyf (2005)	X	X							
Mikulincer and Shaver (2012)							X		
Moher et al. (2015)									X
Monk et al. (2016)				X					
Montirosso et al. (2013)		X	X						
Moore et al. (2016)				X					
Mulder et al. (2021)	X	X							
Mulkey and du Plessis (2019)								X	
Murgatroyd et al. (2015)	X								
Nagy and Turecki (2012)								X	
Norholt (2020)					X				
Norman et al. (2015)		X							
Opendak et al. (2019)								X	
Ortiz et al. (2017)				X					
Packard et al. (2021)		X							
Parr et al. (2022)								X	
Petters and Waters (2014)			X						
Piaget (1936)							X		
Porges and Furman (2011)		X							
Provenzi et al. (2017)	X								
Pun et al. (2021)			X						
Rakers et al. (2017)				X	X				
Rasmussen and Storebø (2021)	X								
Robakis et al. (2020)	X			X					
Roisman and Fraley (2008)	X								
Sai (2005)						X			
Salihagic Kadic et al. (2009)					X	X			
Sandman et al. (1997)					X				
Schindler (2019)							X		
Schore and Schore (2008)		X					X		
Schwab and Rakers (2022)				X					
Seckl and Holmes (2007)				X					
Shapiro and Spaulding (2021)								X	
Sheinbaum et al. (2015)							X		
Sherman et al. (2015)			X						
Simmons (2011)				X					
Smorti et al. (2020)							X		
Sonne et al. (2023)			X						
Sosnowski et al. (2018)				X					
Southgate et al. (2008)			X						
Spangler et al. (2009)	X								
Sroufe (1996)							X		
Sroufe and Waters (1977)							X		
Stauffer et al. (2020)							X		
Streri et al. (2013)						X			
Sullivan and Opendak (2020)								X	
Suzuki et al. (2012)	X								
Teng et al. (2020)							X		
Thaler et al. (2020)	X								
Thompson (2021)							X		
Tribe et al. (2018)				X					
Uchida-Ota et al. (2019)					X				
Vaglio (2009)					X				
Van den Bergh et al. (2020)				X	X				
Van Hoof et al. (2019)		X							
van Ijzendoorn and Bakermans-Kranenburg (2006)	X								
Van Ijzendoorn et al. (2010)	X								
Vöhringer et al. (2018)			X						
Vrticka and Vuilleumier (2012)		X							
White et al. (2020)		X							
White et al. (2023)		X							
Yehuda et al. (2014)				X					
Zietlow et al. (2019)					X				
Zimerman and Doan (2003)							X		

This table details the selection criteria for the 157 studies included in our analysis, which explore the origins of individual differences in the development of the attachment system, starting from the prenatal period. We included articles addressing genetic (GEN), neurobiological (NB), and cognitive (COG) aspects. The analysis encompasses both research directly related to the attachment system and studies on related systems and processes, such as emotional regulation and cognitive functions, including memory and attention. Most of the studies are experimental and review-based, with a particular focus on the first two years of life (INFANCY), extending also to studies that consider wider age ranges but have implications for examining the infant precursors of attachment. The same scientific perspectives (GEN, NB, COG) are also considered for analyzing the literature on attachment-related systems in fetuses and newborns (PERI-NATAL). Additionally, works pertaining to the history and some developments of attachment theory are considered to construct a theoretical framework. The research is supported by multidisciplinary contributions to support the hypotheses relevant to this work. Lastly, an article strictly related to methodological issues is included.

## 4 Attachment and genetics

Genetics is a field within the realm of biology that focuses on investigating the transmission of inherited characteristics from one generation to another. Such an approach is of interest for the purposes of this paper as it could elucidate the weight of innate characteristics on attachment pattern definition. Also of importance is the understanding of the dynamics by which social experiences affect gene expression, being imprinting as a kind of molecular memory capable of influencing later developmental stages.

Attachment could be considered as an innate trait that has survived evolution as it increased our ancestors’ chances of survival (Bowlby, 1969/1982). As an instinct, it probably depends on genetic drives, but the characteristics of the social environment to which the individual is early exposed appear to be the most relevant factor for the definition of the quality of an attachment relationship. The search for the genetic origins of attachment is relatively recent and in the last decades behavioral genetics, molecular genetics, gene by environment association studies and epigenetics have constituted the main approaches to the problem of the hereditary component of this complex construct (Bakermans-Kranenburg and Van IJzendoorn, 2016). In the context of behavioral genetics, studies on groups of monozygotic and heterozygous twins have brought contrasting results (Bokhorst et al., 2003; Donnellan et al., 2008; Roisman and Fraley, 2008) which, all in all, attribute no value to genetics, with respect to environmental variables, in the explanation of the variance of different attachment patterns in children. Along stages of life, the genetic component becomes progressively more valuable (Erkoreka et al., 2021). Fearon and colleagues (Fearon et al., 2014) in a twin study conclude that genetics accounts for a greater and relevant variance (35%) of the security in attachment during adolescence. Molecular genetics has explored the issue from a more granular point of view, observing the contribution of single genes or genetic patterns in the definition of the complex attachment phenotype without reaching, for now, the discovery of one or more “attachment genes” (Bakermans-Kranenburg and Van IJzendoorn, 2016). Research examining the interplay between genes and the environment has yielded intriguing findings, with ample evidence demonstrating the existence of genetic factors that can increase the risk or susceptibility to the influence of environmental conditions on attachment dimensions. Epigenetics is another promising approach. It concerns the specific study of the effects of environmental exposures (as well as other factors such as age) on biological processes such as methylation, a particularly relevant process in behavioral genetics that alters DNA expression by adding a CH3 group to promoter sites of a gene, that impairs the ability of a gene to be expressed and thus to provide instructions for the production and dosage of certain molecules. The approaches that have included environmental influence are the ones that have brought the most evidence and plausibly will be further explored in future research (see Table 2).

**Table 2:** Attachment and genetics.

**Behavioral genetics**	Studies on twins in order to comprehend the impact of Genetics w.r.t. that of the Environment to define specific Attachment dimensions (e.g., Safety)	The weight of Genetics w.r.t. Environment is low but it increases (up to 35%) with the age		Bokhorst et al. (2003); Donnellan et al. (2008); Roisman and Fraley (2008); Erkoreka et al. (2021); Fearon et al. (2014)
**Molecular genetics**	Single genes or genetic pattern	Complex Attachment phenotype	Attachment genes	Bakermans-Kranenburg and van Ijzendoorn (2016)
**Gene × environment**	Genetic polymorphisms acting as risk or protection factors in the definition of specific Attachment patterns. In specific environmental contexts (e.g., not very sensitive parents) there are polymorphisms that act as vulnerability factors in such a way to amplify the effect of the environment			Kochanska et al. (2009); Barry et al. (2008); Bakermans-Kranenburg et al. (2012); Spangler et al. (2009); Gilissen et al. (2008); Hygen et al. (2014); Luijk et al. (2011); van Ijzendoorn and Bakermans-Kranenburg (2006); Gervai et al. (2007); Bakermans-Kranenburg et al. (2011); Suzuki et al. (2012); Erkoreka et al. (2021); van Ijzendoorn et al. (2010)
**Epigenetics**	Effect of environmental exposures on methylation	Biological alteration of DNA	Impairment of gene expression	Rasmussen and Storebø (2021); Craig et al. (2021); Hurlemann and Scheele (2016); Provenzi et al. (2017); Jiang et al. (2019); Robakis et al. (2020); Thaler et al. (2020); Ein-Dor et al. (2018); Ebner et al. (2019); Lecompte et al. (2021)

Various branches of genetics have significantly contributed to the comprehension of attachment development from a biological standpoint. The genetic component plays a crucial role in shaping attachment dimensions, while experiences modulate gene expression through epigenetic processes.

Two recent literature reviews (Craig et al., 2021; Rasmussen and Storebø, 2021) regarding epigenetics and attachment have highlighted that the study of biological alterations of DNA could be of importance for the understanding of the processes that come into play in the interaction between genetics and attachment-related experiences. Craig et al. (2021) provide a systematic literature review analyzing 13 recent experimental works that considered one or more attachment dimensions in relation to changes in the methylation of specific DNA regions. The study brings together evidence that sheds light on the role of methylation of the following genes: OXTR, a gene that codes for the oxytocin receptor and that is involved in the response to social stress and in socio-emotional behaviors such as in affiliative behaviors (Hurlemann and Scheele, 2016); FBPK5, which codes for a protein for the reception of glucocorticoids and which is involved in the stress response; SLC6A4 for the serotonin transporter, which is implicated in stress response processes and socio-emotional functions; HTR3A, which codes for the serotonin receptor and is implicated in emotion regulation; NR3C1, encoding the glucocorticoid receptor that has an important role in the functioning of the hypothalamic–pituitary adrenal axis (HPA). Based on statistical analyses, the authors suggest that it is not possible to establish a cause-and-effect relationship between epigenetic processes and attachment dimensions, however, the correlation is strong and this indicates that the interaction between genetic factors, epigenetic processes and environmental factors can act as predictive indicator of interindividual differences in attachment.

Rasmussen and Storebø (2021) addressed a review of the scientific literature with similar objectives, also highlighting the role of methylation of the gene encoding BDNF (brain-derived neurotrophic factor), a protein implicated in sustaining and protecting neurons from stress-related damage, in the relationship between environmental events and epigenetics. In line with the work of Craig et al. (2021), the authors state that it is possible to consider that the development of stress response systems is subject to the influence of both genetic and environmental factors (particularly, social environment), and that it is mediated by epigenetic processes. Genes involved in attachment bonding are not specific, as they do not uniquely explain attachment-related variables. Rather, they underlie a range of functions, most often of a socio-emotional and nature and they are often involved in stress response and regulation (see Table 3).

**Table 3:** Social environment and epigenetic.

**OXTR**	Oxytocin receptor	Social stress and socio-emotional behavior, affiliative behavior	Hurlemann and Scheele (2016); Rasmussen and Storebø (2021); Craig et al. (2021); Ortiz et al. (2017); Robakis et al. (2020); Thaler et al. (2020); Ein-Dor et al. (2018); Lecompte et al. (2021)
**FBPK5**	Protein reception of glucocorticoids	Stress response	Rasmussen and Storebø (2021); Craig et al. (2021)
**SLC6A4**	Serotonin transporter	Stress response and socio-emotional behavior	Rasmussen and Storebø (2021); Craig et al. (2021); Provenzi et al. (2017); Linnér et al. (2020)
**HTR3A**	Serotonin receptor	Emotion regulation	Rasmussen and Storebø (2021); Craig et al. (2021)
**NR3C1**	Glucocorticoid receptor	HPA functioning, serotonergic system	Rasmussen and Storebø (2021); Craig et al. (2021); Yehuda et al. (2014); Conradt et al. (2018); Sosnowski et al. (2018); Bosmans et al. (2018); Murgatroyd et al. (2015)
**BDNF**	Brain-derived neurotrophic factor	Neuron stress related damage; Sensitivity to parent style	Rasmussen and Storebø (2021); Craig et al. (2021)
**HSD11B2**	Placental enzyme, transforms cortisol into inactive cortisone	Stress response	Monk et al. (2016)

The genes listed in the table are those that are most significantly affected by early social experiences through epigenetic processes. These genes play a crucial role in the activation of the attachment control system.

Going into the subject of what happens at a genetic level during the organization of attachment dimensions, it must be premised that the period from the prenatal phase to the first years of life is to be considered as a particularly important evolutionary window for epigenetic reprogramming and the quality of exposure to the environment and therefore the quality of maternal care is of paramount importance in individual development, with consequences throughout the life course (Nagy and Turecki, 2012).

The fetal programming hypothesis (Seckl and Holmes, 2007) deals with the long-term effects of exposure to environmental perturbations that have the potential effect of influencing the development and organization of biological systems with long-term consequences. In fact, during gestation, the organism prepares to adapt to specific environmental conditions, calibrating the set-points of basal activity, responsiveness and reactivity and influencing the quality of multi-level development. The fetal programming has a lot to do with epigenetics as organizing mechanism. The hypothesis of Developmental Origins of Health and Disease (DOHaD) (Barker, 1990; Barker and Godfrey, 2004) suggests that an individual’s vulnerability to specific physical and mental health issues may be influenced by a blend of genetic factors, environmental conditions during gestation and postnatal environment. During this period, maternal nutrition (Simmons, 2011), maternal secretion of hormones and toxins linked, for example, to high levels of stress or to the intake of substances impact the fetus epigenetic organization, with effects on physical and mental health (Forray, 2016; Schwab and Rakers, 2022). It is known that the early formation of neuronal networks is genetically programmed but epigenetic and environmental influences can modify the course of this process (Linderkamp and Linderkamp-Skoruppa, 2021). In particular, both physical and emotional maternal stress during gestation have an extremely relevant impact. In fact, under stressful conditions, changes in maternal HPA axis take place affecting the transmission of biological information to the fetus via the placenta and umbilical cord (Rakers et al., 2017; Van den Bergh et al., 2020). The effects on the “programming” of the fetus concern the risk for neurodevelopment impairment, the development of anxiety, depression and difficult temperament, autism, schizophrenia and ADHD (Franklin et al., 2012; Van den Bergh et al., 2020), as well as the development of physical pathologies related to cardiovascular functioning, glucose metabolism and body mass (Barker and Osmond, 1986; Barker et al., 1993). Among the target mechanisms for epigenetic modification is stress responsiveness based on HPA axis functioning. Furthermore, as a result of the transgenerational transmission, epigenetic modifications that have occurred in the parents in the face of highly impactful events can be inherited by the offspring without having direct experience of them, with consequences also on attachment (Fujiwara et al., 2019). Among the genes implicated in the transmission of epigenetic alterations is the NR3C1 gene for glucocorticoid receptors (Yehuda et al., 2014; Conradt et al., 2018; Sosnowski et al., 2018). The same gene was found to be involved in the attachment process as children who had this highly methylated gene and experienced less supportive maternal care had high levels of attachment anxiety (Bosmans et al., 2018). During gestation, HSD11B2, a placental gene which codes for a placental enzyme, is implicated in epigenetic processes. It transforms cortisol into inactive cortisone, acting as a protective effect against the effects of stress. Monk et al. (2016) showed that high levels of stress in the mother correlated with methylation of the HSD11B2 gene, resulting in increased exposure of the fetus to stress hormones which negatively affected neurodevelopment.

In the moment of labor that precedes and induces birth, the child undergoes strong mechanical and hormonal stimulation which, according to The Epigenetics Impact of Childbirth (EPIIC) hypothesis (Dahlen et al., 2013) probably has an effect on epigenetic programming (Assmann, 2020). According to the EPIIC hypothesis, the hormonal stimulations that characterize cesarean and natural delivery have recognizable epigenetic effects. In the case of cesarean delivery, drugs, external mechanical maneuvers, and abnormal exposure to intense maternal hormonal secretion has a long-term effect on the functioning of the baby’s HPA axis (Tribe et al., 2018). Among the genes implicated in differential methylation, there is OXTR (Ortiz et al., 2017) for the oxytocin receptor, already mentioned as a gene implicated not only in the stress response but also in attachment dimensions.

Starting from the first hour after childbirth (Moore et al., 2016), the newborn’s direct contact with the mother and breastfeeding exerts a significant influence on epigenetic modulation (Linnér et al., 2020). Skin-to-skin contact at birth, exerts and effect on vagal regulation and serotonin secretion in the newborn, with consequences on glucose levels regulation, precocity of attachment to the mother’s breast, thermoregulation, heartrate stability and stress regulation (Assmann, 2020). In later stages, an extremely important factor is breastfeeding. When this is early and sustained, positive effects on physical health and neurodevelopment can be observed. Epigenetic processes mediate the relationship between these early events and long-term effects. In particular, it emerges that the methylation of NR3C1 (Murgatroyd et al., 2015) and SLC6A4 genes (Provenzi et al., 2017; Linnér et al., 2020), plays an important role for in organizing the serotonergic system and the HPA axis of the individual, with an impact on the predisposition to a certain quality of attachment.

In subsequent periods, the factors that most influence epigenetic processes are related to the quality of care by the primary caregiver: neglect, abuse and variations and the quality of care have a particularly intense effect (e.g., Provenzi et al., 2017; Jiang et al., 2019). Maternal sensitivity over the first months of life influences the methylation of the SLC6A4 gene, with effects on socio-emotional abilities of the infant (Provenzi et al., 2017) and on attachment bonding, and the OXTR gene, encoding the oxytocin receptor, has a role in the development of attachment as its methylation has been traced in subjects with insecure attachment in both clinical (Robakis et al., 2020; Thaler et al., 2020) and non-clinical clinical population, particularly with avoidant insecure attachment (Ein-Dor et al., 2018). Conversely, subjects with lower gene methylation have lower levels of anxious attachment (Ebner et al., 2019). Furthermore, low gene methylation in children has shown negative correlations with maternal caregiving behaviors (Lecompte et al., 2021). It is therefore to be concluded that environmental factors have a fundamental importance (see Table 4).

**Table 4:** Epigenetic and its impact on organization.

**Maternal nutrition and hormones secretion, environmental perturbations during pregnancy** (DOHaD and fetal programming hypotheses)	NR3C1 HSD11B2	Related to the organization and development of biological systems in the fetus and on health outcomes over life	Impacts fetus and baby’s neurodevelopment, temperament, body mass. Related to later presence of Anxiety; Depression; Autism; Schizophrenia; ADHD; Cardiovascular pathologies	Seckl and Holmes (2007); Barker (1990); Barker and Godfrey (2004) Simmons (2011); Schwab and Rakers (2022); Forray (2016); Linderkamp and Linderkamp-Skoruppa (2021); Rakers et al. (2017); Van den Bergh et al. (2020); Franklin et al. (2012); Barker and Osmond (1986); Barker et al. (1993); Fujiwara et al. (2019); Yehuda et al. (2014); Conradt et al. (2018); Sosnowski et al. (2018); Bosmans et al. (2018); Monk et al. (2016)
**Childbirth** (EPIIC hypothesis)	OXTR	Relevance of drugs assumption during delivery, cesarean *VS* natural delivery, Mechanical manoeuvres, massive maternal hormonal perturbations	Effects on the development of baby’s HPA axis	Dahlen et al. (2013); Assmann (2020); Tribe et al. (2018); Ortiz et al. (2017)
**Newborn’s direct contact with the mother**	NR3C1 SLC6A4	Impacts baby’s vagal regulation and serotonin secretion	Effects on baby’s glucose level regulation; precocity of attachment to mother’s breast	Linnér et al. (2020); Murgatroyd et al. (2015); Provenzi et al. (2017)
**Breastfeeding**	NR3C1 SLC6A4	Impacts on baby’s thermoregulation, heart-rate stability, shared regulation	Effects on newborn’s physical health and neurodevelopment	Moore et al. (2016); Assmann (2020)
**Quality of care**	SLC6A4 OXTR	Maternal sensitivity in the first month showed to be important	Effects on socio-emotional abilities	Provenzi et al. (2017); Robakis et al. (2020); Thaler et al. (2020); Ebner et al. (2019); Lecompte et al. (2021)

During the perinatal period, the adaptation (social) environment impacts both psychological and biological organization through epigenetic adjustments. The table presents the events that exert epigenetic influence on genes previously identified as implicated in attachment. It is hypothesized that the social environment (and especially the mother) plays a role in the formation of this bond starting from the prenatal period.

However, it is necessary to consider that certain genetic polymorphisms also contribute to the formation of specific developmental trajectories. What follows is a description of genes that have been identified as risk/protection or susceptibility genes during the complex process of maturation of attachment dimensions. In other words, some evidence highlights that certain genetic characteristics mediate the relationship with the environment, influencing the outcome. A study by Kochanska et al. (2009) on preschool children reported that the 5HTTLPR gene, when in the presence of one or two short alleles, was a risk factor for the development of poor self-regulatory skills in the presence of insecure attachment. This result has been replicated in other studies, including that of Barry et al. (2008) that considered a sample composed of mother–child dyads, and from which it emerged that children who at 7 months are exposed to insensitive maternal care and who possessed a short allele of the 5HTTLPR gene, were more likely to develop at 15 months an insecure attachment style compared to children exposed to sensitive maternal care having the same genotype. In the same study, the presence of two long alleles of the 5HTTLPR gene correlated with a greater likelihood of secure attachment, regardless of the degree of maternal sensitivity. In contexts of strong affective deprivation, such as those of institutionalization, the long variant of the gene keeps its protective effect (Bakermans-Kranenburg et al., 2012). In the presence of this variant, disorganized attachment was not found even in cases of exposure to affective deficiency. Other studies have confirmed the risk or protective factor value of the two variants of the 5HTTLPR gene in the mediation between attachment security as an outcome and maternal reactivity as a predictor (Spangler et al., 2009) and in the mediation between the ability to regulate emotions in stressful conditions as an outcome and attachment security as a predictor (Gilissen et al., 2008).

The COMT gene, which has a fundamental role in dopamine metabolism, has also been shown to play a role in mediating the relationship between attachment and environmental variables. In cases of disorganized attachment, children with the Val polymorphism of the gene have more aggressive and antisocial behaviors than children who have the same attachment but possess at least one Met allele of the gene (Hygen et al., 2014).

In a 2010 study by Luijk et al. (2011) also the FBKP5 gene, which codes for the homonymous protein involved in cortisol reactivity, was shown to play a role in the mediation between attachment as a predictor and cortisol reactivity as an outcome: 15-month-olds babies with one or two T alleles of the gene and insecure attachment, showed an elevated risk of cortisol reactivity.

The DRD4 gene, for the D4 dopamine receptor, is among the most significant genes in the study of developmental genetics. DRD4 is regarded as a susceptibility factor, meaning that the presence of a certain polymorphism of the gene functions as an amplifier of the effect of environmental conditions, whether favorable or unfavorable. Specifically, the 7-repeation DRD4 has been shown to function as a differential susceptibility gene with the following results (van Ijzendoorn and Bakermans-Kranenburg, 2006): in the presence of the 7-repeation polymorphism and early exposure of the child to a mother with unresolved trauma, the probability of developing a disorganized attachment notably increased. When children without 7-repeation genes were exposed to similar maternal conditions there was no increased likelihood of having disorganized attachment. On the other hand, when children with DRD4 at 7 repetitions were cared for by mothers who did not present trauma or unresolved losses, the lowest probability of disorganized attachment was observed. Subsequent studies confirmed the hypothesis of DRD4 as a susceptibility factor (Gervai et al., 2007; Bakermans-Kranenburg et al., 2011).

Among susceptibility genes, also BDNF appeared to be involved in adults (Suzuki et al., 2012; Erkoreka et al., 2021). In fact, the presence of polymorphisms in the gene encoding for BDNF that contain at least one Met allele correlates with higher sensitivity to parent styles and environmental contingencies.

From the Generation R study which considered over 500 dyads extracted from the Rotterdam population, it emerged that the genes coding for glucocorticoid (GR) and mineral-corticoid (MR) receptors are also involved as susceptibility factors in the organization of an attachment style. These receptors play a role in regulating the stress response. Luijk et al. (2011) showed how the MR receptor in the polymorphism presenting at least one minor *g* allele mediates the relationship between maternal sensitivity and attachment security. Children having the aforementioned polymorphism are more prone than average to develop secure attachment in case of sensitive maternal care and they are more likely to develop an insecure attachment to insensitive mothers (see Table 5).

**Table 5:** Gene × environment: risk and susceptibility factors.

**Risk/protection factors (A)**			
**5HTTLPR**	Short allele/s polymorphism	Poor self-regulatory skills in the presence of insecure attachment Risk for insecure attachment in case of maternal insensitivity	Kochanska et al. (2009); Barry et al. (2008); Bakermans-Kranenburg et al. (2012); Spangler et al. (2009); Gilissen et al. (2008)
	Long allele/s polymorphism	Higher likelihood of secure attachment regardless of the caring environment	
**COMT**	Val allele/s polymorphism	Aggressive and anti-social behavior in case of disorganized attachment	Hygen et al. (2014); Luijk et al. (2011)
**FBKP5**	T allele/s polymorphism	In case of insecure attachment predicts cortisol reactivity	Luijk et al. (2011)
**Susceptibility factors (B)**			
**DRD4**	7 repetition polymorphism + compromised maternal caring	Increased probability of attachment disorganization	van Ijzendoorn and Bakermans-Kranenburg (2006); Gervai et al. (2007); Bakermans-Kranenburg et al. (2011)
	7 repetition polymorphism + NOT compromised maternal caring	Decreased probability of attachment disorganization	
**Gene for GR and MR receptors**	g allele/s polymorphism + sensitive care	Increased probability of secure attachment	Luijk et al. (2011)
	g allele/s polymorphism + sensitive care	Increased probability of insecure attachment	
**BDNF**	Met allele/s polymorphism	Increased sensitivity to parent style	Suzuki et al. (2012); Erkoreka et al. (2021)

The behavioral systems used by attachment are innate and genetically predisposed, and the specificity of attachment development is attributed to the quality of the interaction experience with the caregiver. The table presents the genetic polymorphisms that act as risk **(A)** or susceptibility **(B)** factors for the characterization of the dimensions that are used by the attachment system.

The effects of candidate genes on attachment which were discussed above can be mitigated by epigenetic changes. The protective role of the 5HTTLPR gene in long polymorphism is lost when following repeated negative experiences in case of hypermethylation. Similarly, the short version of the gene loses its impact of risk factor when methylated (van Ijzendoorn et al., 2010). Overall, the effects of epigenetic modifications are long-lasting and act as a cellular memory. However, they can be counteracted by pharmacological actions or strong experiences as a product of memory and learning (Champagne, 2010).

## 5 Attachment and neurobiology

The section aims to provide an overview of neurobiological mechanisms that have been observed in relation to attachment dynamics. In the process of review, we included gestation as we consider it as a relevant period for the understanding of attachment-related neurobiological variations. The neurobiological organization of the individual consists of multiple interacting organic systems (e.g., HPA system, immune system, neural networks) whose organization influences and is influenced by significant affective events, especially when these occur early in the individual’s development. The attachment behavioral system is useful in maintaining physical and felt security, and its activation follows the emergence of disturbances to psychophysical balance (Bowlby, 1969/1982). From a neurobiological point of view and particularly from the perspective of social neuroscience of human attachment (Coan, 2016; Feldman, 2017; White et al., 2023), the main function of attachment represented by security is closely related to organic homeostasis In this sense, the caregiver is necessary to the infant for co-regulation, this is when disturbances to a homeostatic state are managed through social-allostasis (Coan and Sbarra, 2015; Atzil et al., 2018). The caregiver serves as good regulator as long as he or she is able to cooperate with the infant in restoring homeostatic conditions, minimizing the energy the infant must expend in doing so (Atzil et al., 2018). This hypothesis is consistent with attachment theory, which observes interindividual differences in the development of attachment patterns as a function of the infant’s ability to find a secure base in the parent’s proximity. Accordingly, Schore and Schore (2008) referred to attachment theory as a theory of regulation, as developments in the field have shed more and more light to the security of attachment in the programming of the child’s physiological systems underlying nervous system regulation. Attachment style reflects the capability for co- and then self-regulation of internal states. During development, dependency on co-regulation decreases (Atzil et al., 2018); however, the buffering effect generated by the presence or mental representation of a caregiver (Norman et al., 2015; Long et al., 2020) acts as a buffer for the restoration of internal equilibrium (Packard et al., 2021). The caregiver’s buffering function has been confirmed by studies on animal models (Opendak et al., 2019; Sullivan and Opendak, 2020).

In light of what has been described so far, we support the hypothesis of other authors (e.g., Atzil et al., 2018; Long et al., 2020; White et al., 2020) that attachment may not be an innate instinct but a learned process given by the necessity of social allostasis and that various phenotypes are a product of allostatic regulation strategies’ maturation. However, we maintain that the prenatal period is of considerable importance, and that social learning processes are already underway during this period through sensory and organic stimuli. In this regard, Ciaunica et al. (2021) have described the need for typical allostatic co-regulation during the gestational period, when mother and baby inhabit the maternal body together. In this condition of co-embodiment, continuous negotiation occurs between mother and fetus for the maintenance of the homeostatic balance of both organisms and their survival. The fetus’ nervous system learns from environmental statistics (high or low cortisol levels transmitted by mother? Physical conditions, such as the sound of mom’s heartbeat, comfortable or uncomfortable?) to calibrate homeostatic values whose disruption will trigger a stress response. According to an embodied approach, learning of cognitive and perceptual schemas maintains the body schema as the nuclear (Ciaunica et al., 2021). Thus, the quality of intrauterine experience, being the first experience, generates “proto-social” learning through organic and sensorial stimuli, which lay the basis for all subsequent acquisitions. The persistent condition of maternal stress during pregnancy is a concrete example of how prolonged alteration of maternal homeostasis affects fetal organization with significant consequences for the cognitive, social, and emotional development of the child after birth, mediated by the shaping of the HPA system and the autonomic nervous system (Van den Bergh et al., 2020). Recently, the mother’s emotional state during pregnancy has been associated with the fetus’s attention and arousal abilities as factors that mediate attachment security 10 months after birth (DiPietro et al., 2021). Moreover, external stimuli such as mothers’ speech during pregnancy have been demonstrated to be pivotal in the maturation of bilateral fronto-temporal networks, areas implicated in language and voice processing (Uchida-Ota et al., 2019) but also in social and attachment related functions (Atzil et al., 2011). Examples of the fetus’ adaptive attunement based on information from the mother could be the organic substrate of internal representations of the world, and it is given by the necessity to maximize the probability of survival during gestation and with a view to the future extra-uterine life. Prenatal exposure to the mother’s voice and the co-homeostatic environment *in utero* contribute significantly to the acquisition of social competence in newborns. Therefore, it is possible that social competence is not innate but rather learned since pre-natal, as the fetus acquires the capacity to process and responds to social cues through interactions with the mother’s physiology and auditory (but also tactile) stimulations.

According to a neuroanatomical perspective, Long et al. (2020) provided an insightful functional model of attachment (NAMA), a theoretical framework that draws on both first-person and second-person social neuroscience to explain neural correlates of regulatory mechanisms in secure and insecure organized attachment patterns. First-person social neuroscience refers to the study of brain activity in response to internal stimuli, such as emotions, thoughts, and bodily sensations. Second-person social neuroscience, on the other hand, focuses on how the brain processes social interactions and relationships with others.

Firstly, the authors (Long et al., 2020) review attachment pathways illustrating how secure, insecure avoidant, and insecure ambivalent attachment differ in their response strategies to stressful events that disrupt the homeostatic condition and require targeted operations for allostatic self- or co-regulation. Achieving physiological regulation corresponds to a sense of security, as well as social reward when the homeostatic condition is achieved with the help of a caregiver. The repetition of these cycles leads to the establishment of IWM about oneself and the world. In the case of secure attachment, proximity to the caregiver is used as a strategy for emotional and allostatic co-regulation and systematically corresponds to actual restoration of the homeostatic condition and a sense of security that encourages future interactions in times of stress. In the case of secure attachment, even fight or flight responses can be flexibly used depending on the situation and in most cases correspond to physiological and emotional regulation. In the case of organized insecure attachment, secondary strategies are rigidly (versus flexibly) used. Avoidant attachment most often corresponds to the preference for self-regulation strategies with suppression and inhibition of internal states, at the expense of co-regulation strategies. In contrast, anxious attachment sees the preference for proximity seeking for co-regulation to the disadvantage of self-regulation strategies. The main difference between secure and insecure attachment is the marked difficulty, in insecure attachment patterns, of restoring the homeostatic state and experiencing the derivative sensation of security.

Going into the neuroanatomical details of the model and consistently with what has just been described, the authors (Long et al., 2020) indicate the presence of four modules involved in attachment processes, two of which pertain to the affective component, aversion module and approach module, and two of which pertain to the cognitive component, emotion (self-) regulation module and mental states representation module. The activation of one of the affective modules excludes the activation of the other, and the same holds true for the affective system versus the cognitive one. When a potential threat is detected, the aversion module is triggered, resulting in stress and fear responses. The activation of this module is linked to the extended saliency network, which involves several regions, including the amygdala, hippocampus (with consequences on HPA), insula, anterior cingulate cortex, and anterior temporal pole (e.g., Vrticka and Vuilleumier, 2012). The approach module underlies the motivation to seek social proximity as well as the feeling of safety and it contrasts the activation of the aversion module. The ventral tegmental area, substantia nigra, ventral striatum, and ventromedial prefrontal cortex are regions of the brain that play a role in reward processing, primarily through dopaminergic activity. The sense of safety is better represented by the joint action of other neurotransmitter systems, such as vasopressin, oxytocin, opioids, and serotonin (e.g., Feldman, 2017). The emotion (self-) regulation module concerns the functions of voluntary regulation of emotions, also through social behaviors. When this module is activated, it typically results in decreased activity of the aversion module and a reduction in physiological responses that have been previously impacted by stress. Mental states representation is a cognitive function that allows for the creation of predictions and inferences about others’ states, which allow predicting the outcomes of certain behavioral sequences. These are essential features of IWM, derived from repeated experience. The areas indicated by the model as mainly involved are medial orbitofrontal/prefrontal cortex, posterior cingulate cortex, precuneus, superior temporal sulcus, temporoparietal junction, anterior superior temporal gyrus, fusiform gyrus. According to the authors (Long et al., 2020), the activation of attachment pathways begins when the subject comes into contact with an internal or external threat and with the activation of a primary fear response. To this proposal, we add the hypothesis that this mechanism may be functioning, although difficult to observe, during the prenatal period, as the fetus is already able to experience differential emotional states and can be stressed by events that alter its homeostatic state, such as accelerated maternal heart rate, the presence of unpleasant chemosensory substances, excessive movement or certain positions of the mother, and so on. The fetal condition implies continuous at least biological and organic connection to the mother, and it should be investigated whether maternal touch or other specific caregiver-related conditions can be perceived as a specific stimulus capable of activating neuronal patterns similar to those of affective and cognitive modules.

According to NAMA (Long et al., 2020), attachment security coincides with an increase in the activity and efficiency of the (self-) regulation module and a decrease in the aversion module. A better capacity to understand and predict others’ internal states goes along with an enhanced functioning of the mental states representation module in secure attached individuals. An interesting feature of the neurofunctioning of secure versus insecure attachment is given by the increased activity of the ventromedial prefrontal cortex (Eisenberger et al., 2011) and areas involved in reward processes (Choi et al., 2018). With attachment avoidance, the activation of the approach module is altered, so when the attachment figure is not taken into consideration for co-regulation, the proximity of the other can even induce the activation of the aversion module. Mental representations are not used to attenuate the stress response (Norman et al., 2015). In anxious attachment, the greater vulnerability in the activation of the fear response tends to result in hyperactivation of the approach module. Regarding (self-) regulation, in avoidant attachment, the self-regulation strategy prevails, with the use of inhibition or suppression strategies and anomalous activation of the HPA system.

The HPA axis is an important regulatory system which, as we have seen from the literature just reviewed, plays a fundamental mediating role in social development. It regulates stress responses and is instrumental in shaping how individuals respond to stress and form social bonds. In the context of attachment, the functioning of the HPA axis can influence emotional regulation and social behaviors, particularly in response to stress or threat. Abnormalities or variations in HPA axis activity are often associated with differences in attachment styles and can impact an individual’s ability to form and maintain healthy relationships (e.g., Meaney and Szyf, 2005; Mulder et al., 2021). Adverse events experienced in the early years of life, such as exposure to dysfunctional attachment patterns (Jarvis, 2022), have a particular effect on the organization of the HPA axis and other biological regulatory systems (e.g., oxytocinergic, SNA and immune, epigenetic regulation, HPG), involved in the development of the ability to regulate allostatic load. Long-term consequences for psycho-physical health are given by the complex interplay of HPA, autonomic nervous system, and immune system (Cole, 2013; Ehrlich et al., 2016; Epel et al., 2018; Engel and Gunnar, 2020). The period from the prenatal phase to the second year of life is considered to be of particular importance for the programming of HPA system, and in case of excessive exposure to early stress and in the absence of social and/or genetic buffers (e.g., Jinxia and Xuxia, 2022), it may calibrate toward a pattern of activation that results in early hyper or hypo cortisol reactivity (Johnson et al., 2018).

The second-person perspective in the NAMA model is key for understanding social information processing and emotional cues. This includes empathy, theory of mind, and social perception, involving neural processes like mirror neuron system activation for empathy, medial prefrontal cortex for theory of mind, and amygdala and fusiform gyrus for social perception. Additionally, social learning involves the reward system. Behavioral aspects include attention to, interpretation of, and response to social cues, influenced by individual, cultural, and societal factors (Long et al., 2020).

Research suggests that individuals with different attachment styles may exhibit differences in the second-person perspective, or how they perceive and respond to social information and emotional cues from others. Securely attached individuals tend to exhibit high levels of empathy, theory of mind, and social perception, which allow them to effectively understand and respond to social information from others. They are also more likely to engage in positive social learning, as they are able to effectively interpret and respond to social feedback. In contrast, individuals with anxious or avoidant attachment styles may exhibit differences in the second-person perspective that reflect their attachment patterns. For example, individuals with anxious attachment styles may exhibit heightened sensitivity to social cues and emotional information, but may have difficulty regulating their emotional responses. They may also have difficulty accurately interpreting others’ mental states, leading to misunderstandings and interpersonal difficulties. Individuals with avoidant attachment styles, on the other hand, may exhibit lower levels of empathy and social perception, and may be less likely to engage in positive social learning. They may also have difficulty accurately interpreting social feedback, and may be more likely to disengage from social interactions. Overall, while there may be individual differences within attachment styles, research suggests that attachment patterns can have important implications for the second-person perspective and how individuals perceive and respond to social information from others.

In conclusion, at birth, the baby has a well-developed Sympathetic Nervous System, ready to activate the body in case of disturbance. The parasympathetic nervous system, useful for regulating and maintaining a state of balance, is more immature and requires longer development time. Therefore, the presence of a responsive caregiver is necessary for the infant’s regulation minimizing energy dissipation (Porges and Furman, 2011; Atzil et al., 2018; Mulkey and du Plessis, 2019). The infant’s co-regulation given by proximity is well observable since the very first hours after birth (Assmann, 2020) with effects that can be noted in biobehavioral (e.g., sleep rhythms and heart rate regulation), cognitive and psychosocial (e.g., attachment quality) skills (Norholt, 2020). This section discusses the neurobiological mechanisms that underlie attachment dynamics, including during gestation. Attachment style reflects the capacity for co- and self-regulation of internal states, and the presence or mental representation of a caregiver acts as a buffer for the restoration of internal equilibrium. The quality of intrauterine experience is the first experience that generates proto-social learning through organic and sensorial stimuli, laying the basis for subsequent acquisitions. A functional model of attachment has been proposed and involves several neural systems that interact for the formation of attachment styles (Long et al., 2020). Tracking its neurobiological underpinnings is crucial for the understanding of human attachment and interindividual differences along with different social stimuli.

## 6 Attachment and cognition

Cognitive aspects of attachment involve consideration of the ways in which repeated attachment experiences affect processes such as evaluation, perception, attention, memory, and prediction and how they mediate the perpetuation of certain patterns acquired very early in life. In attachment theory, IWM are the mainstay for the study of the cognitive substrate, and in the present section the discussion is focused on this very topic.

IWM contain caregiver-specific relevant information such as where they are in the space and what are expected behaviors (e.g., availability) in certain circumstances (Bowlby, 1973), and they guide perception and evaluation of events (Bowlby, 1980). Bowlby (1980) follows Craik’s (1943) pioneering idea of internal models of the world, claiming that IWM are representation of structural features that are used to analyze, predict and, eventually, prevent attachment-related conditions through optimal adaptive behaviors. An example is given by IWM of predicted caregivers’ unavailability in case of need, which corresponds to child’s preventive behaviors of isolation to avoid rejection during hard times. IWM are adjusted through assimilation and accommodation, as described by Piaget (1936), they are not rigid, but flexible cognitive structures that become more automatic over time to optimize resources, and they exert their influence over thoughts, emotions and behaviors without necessarily reaching consciousness. But how do IWM evolve? A literature review by Sherman et al. (2015) reconstructs this process by combining literature from cognitive and social developmental psychology, aiming at understanding if in the first year of life, children are able to form caregiver-specific and experience-based IWM. What emerges is that the formation of IWM relies on the maturation of certain cognitive skills: recognition of- and discrimination between people, notion of permanence of people, memory of past experiences, creation of expectations based on experience, and understanding of the meaning of caregiver’s behaviors and emotions. These features are difficult to approach experimentally in the first year of life, in fact, the number of studies in this regard is rather small.

Recognition and discrimination of people are early skills, and they are necessary to maintain updated caregiver-specific patterns based on new experiences. They are needed for the “who” component of IWM. The baby immediately after birth is able to recognize the mother’s voice and prefer it over other voices, including female voices. The pairing of mother’s voice and face also enables its recognition by vision. Recognition and discrimination are gradually refined, also influenced by the development of sensory and attentional skills. The notion of permanence of the person concerns the ability to create a representation that endures even when the represented person leaves the field of vision of the subject for prolonged periods of time. Apparently, this capacity appears in the first year of life; in fact, the absence of the caregiver is still informative for the creation of IWM (Bowlby, 1969/1982, 1973). The permanence of the person is important in the construction of IWM as it allows caregivers to be mapped, knowing where they can be found in space.

Memory of past events can be divided into implicit and explicit memory, which differ in certain characteristics such as rigidity, reliability, availability of the memory to consciousness, and speed of acquisition. According to a recent study (Vöhringer et al., 2018), implicit memory emerges early and its maturation is completed around the third month of life. Both processes are, albeit in a prototypical manner, operational during the first year and enable the child to use certain emotional aspects to recall previous interactions. Starting from the sixth month, infants are able to codify the information about dynamical events and to retain the memory over 2 weeks (Sonne et al., 2023). At around 9 to 11 months, they can recollect more intricate events over extended durations with decreased exposure time.

Of particular interest in the study of attachment is long-term affective memory that concerns emotions and significant interactions with others. As shown by an experimental study that considered behavioral and physiological responses (Montirosso et al., 2013) infants are able to retain for 15 days memories of social events that generated a stress response, since their fourth month.

Another important aspect is that of expectations, that have been observed very early in life and are useful in executive functions of IWM. The utility of remembering past events is to track statistics that make it possible to predict future caregivers’ behaviors when needed. In these regards, it is not clear whether the patterns of attachment behaviors observable during the Strange Situation are given by predictions of caregiver’s behavior or by an emotional response whereby, for example, infants do not appreciate the closeness of the detached parent in case of need (Sherman et al., 2015). From 3 months onward the infant reacts differently to facial expressions whether they are made by the mother, the father or a stranger and this is significant referring to social expectations. At 4–5 months of age, infants respond with a higher level of dysregulation when it is the caregiver rather than a stranger to be unresponsive to needs (e.g., Gekoski et al., 1983; Lamb and Malkin, 1986). There is evidence that social expectations could be innate and infants as young as 9 months demonstrated to have expectancies on positive social behaviors while observing conflict in an interactive group of adults (Pun et al., 2021). Other than innate expectations, infants build different predictions based on past experiences with specific adults. For example, infants expect adults to behave in ways that are consistent with past goal-directed interactions. For instance, at 9 months of age, even from neuroimaging observations, it is possible to distinguish between infants who have observed behaviors aimed at a new goal and behaviors aimed at the same goal as a previous observed condition (Southgate et al., 2008). Infants under one year of age can recognize adult preferences and goals through the observation of goal-directed sequences of actions and by using cues of adults’ focus of attention and emotional state and generate expectations about their consistent behavior. Thus, in addition to preferences and past experience of caregivers’ behavior, infants use the detected emotional state to predict the adult’s behavior in novel circumstances.

The last cognitive skill identified by Sherman et al. (2015) as significant for the formation and development of IWM is understanding the caregiver’s behavior and emotions. The emotional response of caregivers to attachment behaviors is informative and contributes to IWM if the child is able to recognize the various expressions by attributing meaning to them. Basic emotions can be recognized even by very young children and this ability refines over time. At 6 months infants can discriminate emotions by using vocal and visual cues and can notice discrepancy between emotional information coming from the two modalities (Palama et al., 2018). Emotion recognition is informative when connected to the interpretation of intentions, goals, predisposition, and future actions of the person who is experiencing them. Between the second and the third month, infants behave differently to maternal emotions of joy, sadness, and anger (Haviland and Lelwica, 1987). Another interesting aspect pointed out by the authors (Sherman et al., 2015) is that infants give valence to ambiguous objects based on the recognized emotional response in the caregiver. At 12 months, the infant prefers objects toward which the adult has expressed positive emotions (Flom and Johnson, 2011). Mentalizing others’ intentions implies the understanding that their behavior is voluntary. Recent experimental work has observed that 6-month-old infants are able to understand when the other is imitating them, and this generates prosocial reactions (Sauciuc et al., 2020). Recognizing imitation is a relevant indicator of the understanding another’s intentionality. In the context of attachment, understanding the intentionality of the caregiver’s gesture of uncaring could be particularly important for the formation of IWM. Indeed, it is different whether the parent’s failure to pick up is seen as intention or randomness (Sherman et al., 2015).

Based on experimental evidence reviewed by Kadic and Kurjak (2018), it is hypothesized that some of the cognitive skills examined by Sherman et al. (2015) may exist in a basic form from gestational age, and this leads to our hypothesis that prototypical IWM could precede birth. From the first weeks of gestation, the fetus has surprising skills regarding perception, memory, action, and emotions. Regarding perception, from the work of Kadic and Kurjak (2018) we know that the sense of touch is an early acquisition. At the seventh gestational week, the fetus responds to tactile stimuli with reflexes to nociceptive stimuli and can perceive pain that is processed at a cortical level from the 25th week on. Around the 25th week of gestation, the fetus sensory-motor adjustments lead to body awareness that has been associated with the emergence of consciousness (Lagercrantz, 2007). Chemoceptive senses develop from the 20th gestational week and the fetus shows preferences and differential behavioral responses to tastes after 29 gestational weeks. Regarding hearing, the familiar voices appear to be recognized, and a behavioral preference (measured through signals such as fetal heartrate) is shown for the maternal voice by the 36th gestational week. Vision appears around the 26th week. The development of this modality continues until the eighth postnatal month, and around 36 weeks visual stimuli begin to be processed at the cortical level (for further discussion see Kadic and Kurjak, 2018). All the abovementioned senses could be used by the fetus to gather information about the adaptation social environment.

Two aspects highlighted by Kadic and Kurjak (2018) and of great interest for the study of attachment are fetal emotions and memory. Facial expression and specific behavioral pattern that can be observed during gestation could be indicative of the presence of basic emotions and an awareness condition. These patterns were observed starting from the third trimester in response to maternal voice and preferred tastes (Delafield-Butt and Trevarthen, 2013). Among the main areas involved in emotional memory, emotional recognition and subjective experience of emotions such as fear, pleasure and joy, is the amygdala, that is present during fetal age and completes its maturation during the first year of life. Through habituation experiments, memory of vibro-acoustic stimuli in fetuses has been observed at 22 weeks. Maternal depression and stress worsen fetal development, delaying the emergence of these memory and learning abilities. In stimulus pairing tasks, learning emerged between weeks 32 and 26. In exposure learning tasks, fetuses showed familiarity with stimuli presented numerous times around week 37. The fact that infants are able to discriminate and prefer mother’s voice over others is symptomatic of their being able to learn and potentially use their emotional memory for orientation in extrauterine life, with the possibility of retaining memories up to 6 weeks (Granier-Deferre et al., 2011).

**Figure 5:**
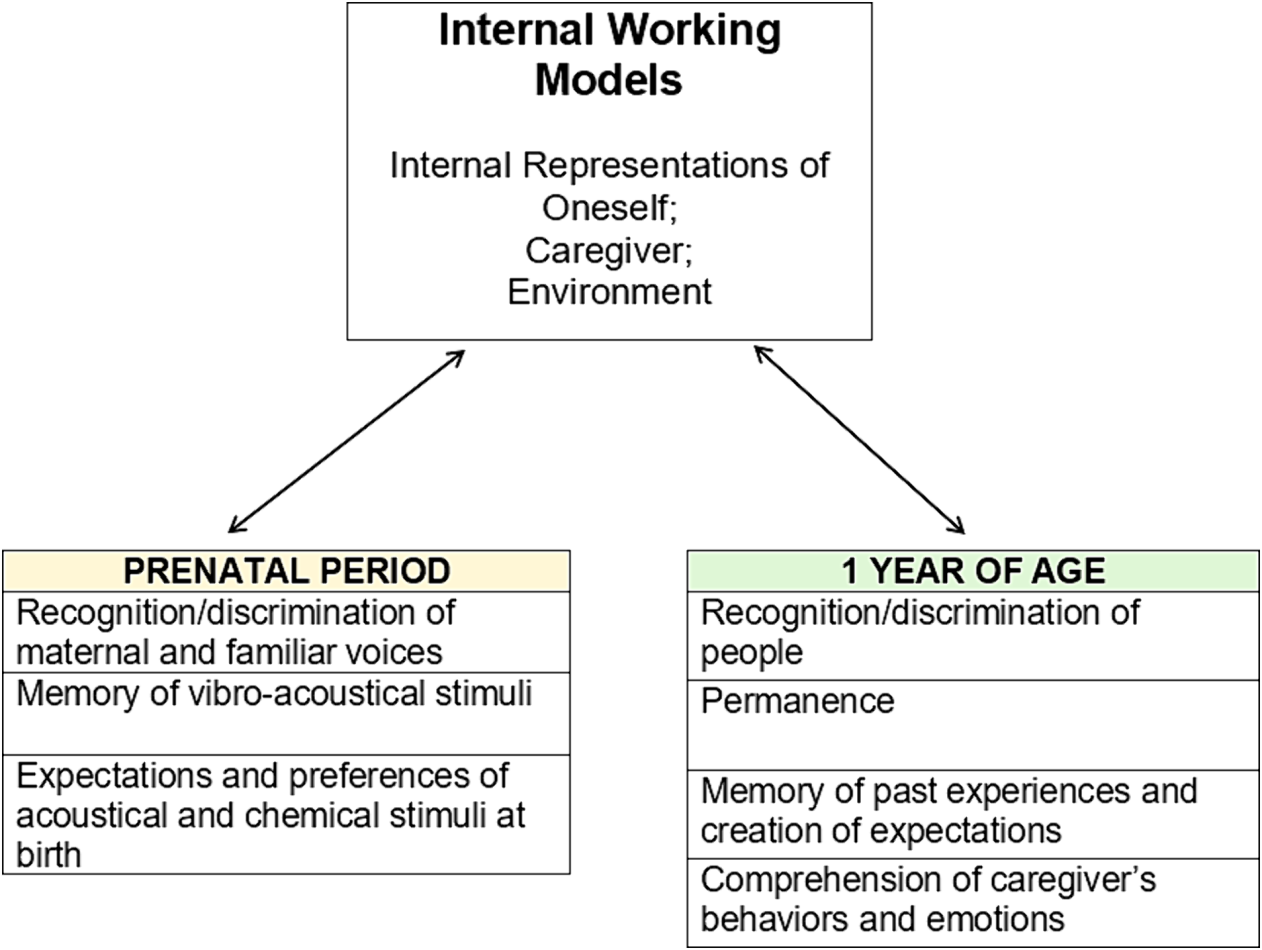
Internal working models are based on interactive experiences with the caregiver, and the hypothesis we support is that the cognitive abilities of the fetus and the child in the first year of life are sufficient to inform and lay the foundation for the attachment model at various levels of complexity.

Prenatal rudimentary learning is considered important for the development of maternal recognition, attachment, and other functions such as social recognition and lactation (Salihagic Kadic et al., 2009) and could play a psychobiological role in areas such as attention and perception (Granier-Deferre et al., 2011) (see Figure 5).

In conclusion, sensory abilities, rudimentary memory skills, and affective life of the fetus, make plausible the speculative hypothesis that experiences with the caregiver influence in specific ways the model of attachment starting from prenatal experience. In his original work, Bowlby (1969/1982) identified one of the fundamental conditions for the success of a working model: the ability to extend itself in imagination to cover potential realities as well as those that are experienced. Therefore, it would not only be plausible but also necessary for IWs to begin their development preceding the need for physical closeness to the caregiver, anticipating it representationally.

An attempt to broaden knowledge of IWM of attachment is the Learning Theory of Attachment of Bosmans et al. (2020), which adds notions from behaviorism to the more traditional treatment of IWM. According to the theory, IWM are acquired through operant and classical conditioning and secure attachment is a “safety conditioning process” that is given by a stimulus that becomes a predictor of a condition in which an adverse event will not occur. In case of secure attachment, after the learning process, conditioned stimuli become inhibitors of fear and stress responses. Authors suggest that it is precisely the caregiver that becomes a conditioned stimulus that inhibits fear and stress. Safety conditioning is supported by neurobiological and endocrinological dynamics, in which conditioning is mediated by the action of amygdala, brainstem, dorsal anterior cingulate cortex, and insular cortex. Neural networks are formed during experiences and guide information processing both at an automatic and voluntary level. At the endocrinological level, cortisol is a hormone that facilitates negative stimulus–response coupling that facilitates the occurrence of avoidance behaviors. Dopamine and oxytocin are relevant in extinction and positive stimulus–response matching. Contingency between conditioned or unconditioned stimuli (such as harm) and support or lack of support is a valuable factor that also explains how environmental conditions can interfere in the definition of attachment security. In fact, the occurrence of distressing events, such as work-related stress, can impair the sensitivity of the caregiver to the infant’s need and can result in an intermittent reinforcement of attachment behaviors. Thus, it is not just the caregiving (or neglecting) behaviors that define IWM and security, but also the continuity or intermittence of reinforcement to attachment behaviors. The authors interpret IWM as a script that affects cognitive processing of attachment-related cognition, such as attention direction, interpretation of caregiver’s behaviors, appraisal of caregiver’s availability, and automatic organization of attachment experience (Bosmans et al., 2020).

An interesting approach to infant’s cognition that also explored the concept of IWM is that of embodied cognition. The latter emphasizes the importance of body and environment for cognitive abilities and according to this field of cognitive sciences “the body or the body’s interactions with the environment constitute or contribute to cognition in ways that require a new framework for its investigation. Mental processes are not, or not only, computational processes. The brain is not a computer, or not the seat of cognition” (Shapiro and Spaulding, 2021, p. 1). Starting from the idea that it is necessary to integrate new approaches to the problem of IWM in order to understand the granular dynamics of their formation and development, some authors revisited the concept of attachment IWM according to notions of embodied cognition (Petters and Waters, 2014; Beckes et al., 2015). IWM, in fact, are a not entirely satisfactory explanation of attachment behavior patterns. They are a cognitive and predictive solution that lacks an understanding of a subjective component of experience. How does the child attribute subjective meaning to the attachment experience? IWM are made to explain how the individual comes to make predictions about caregiver’s behavior but not how the way he or she feels during attachment experiences influences the model.

An interesting view given by Beckes et al. (2015) argues that homeostatic systems can act as embodied signals for relation states and that they do not require mediating mental representations. “Such a link between relationship cognition and sensory perception is rooted in an evolved link between energy regulation and social contact” (Beckes et al., 2015, p. 4). In this sense, attachment-related dynamics such as warmth, separation, and proximity can be explained as anchored in physical resources and by the interaction between body, brain, and external environment (Beckes et al., 2015). The authors argue that individual differences in attachment styles should not be conceptualized as representations, but as response tendencies that arise from the person’s unique biological composition and learning history.

Petters and Waters (2014) contributed to the embodied literature of IWM, suggesting that attachment patterns traditionally explained by mental representations can be supplemented with information such as energy, traditionally explained by psychoanalysis as psychic energy. In this perspective, early experiences could influence an individual’s development through a complex functional organization that includes more than just the memory of events. Rather than representations, then, IWM could be conceptualized as constructions or embodied working models. According to the authors (Petters and Waters, 2014), the infant’s body serves as a stimulus to be processed and coupled with stimuli from the external environment. The cognitive component, therefore, is enriched by embodied information about bodily sensations, physiological responses, and computations concerning the physical system that fall within the focus of the attachment control system. To summarize, from the embodied perspective, subjective feelings and attachment episodes could be conceptualized as structurally coupled. The authors also discuss the developmental temporality of embodied WM, since attachment experiences have an impact on different time scales. In the short term, there are alterations given by an altered state of the child and the permanence of the state is mediated mainly by physiological processes such as the permanence of elevated cortisol levels. Another occurrence concerns the case in which the child gives meaning to the experience. In this case, memory processes that take longer come into play. Another temporal level is that of structural change, which takes even longer and that implies change in the architecture of information processing. In the proposal of Petters and Waters, the attachment control system is given by experiences that are structured into particular architectures that are shaped over time and experience. These structures are not fixed but contingent on context because they are primed by external events. An architecture is invoked by precise stimuli, and what changes are the states accessible from the current state.

Atzil et al. (2018) add to the growing body of research on embodied cognition and its role in shaping human cognition and behavior, highlighting the embodied nature of social cognition. The paper emphasizes the importance of sensory input and physiological regulation in the development of social concepts and competencies. The authors argue that learning in the context of allostasis is a fundamental aspect of social cognition, and that social concepts and skills are acquired through the embodied experiences of synchrony, joint attention, and other social interactions. Infants gain experience interacting with their caregivers, and most interactions are aimed to allostasis regulation. Repeated patterns of sensory information are categorized in statistic predictions about allostasis. Social competencies, such as joint attention and biobehavioral synchrony, are learnt at the service of allostatic needs. The “statistics” of regulating capacity of caregivers is the font from which infants learn social and self-regulation, especially during the first year of life

It is concluded that the child is plausibly able to construct representations due to cognitive development in the first year of life. We hypothesize that such models begin formation even before birth. Mental representations may be an insufficient concept to explain the complex dynamics underlying the way early experiences, child-specific characteristics and environmental events coincide in shaping the organization of the individual. It is possible that an embodied perspective could provide insight into the manner in which certain acquisitions are given meaning by sensory and metabolic information. We argue that it is possible to hypothesize that attachment and its embodied patterns begin their evolution at this very time based on the neurochemical and sensory information with which they come into contact.

## 7 Discussion

The paper analyzes with a close focus the mechanisms through which the attachment experiences that take place until the second year of life are memorized in the individual’s organism at a multi-system level, exerting an influence on its organization that is relatively resistant to change over time.

Attachment is one of the foundational themes in the history of (at least psychological) development of human beings. For this reason, we assume that it must be approached taking into account multiple scientific viewpoints that we have named “perspectives.” The perspectives we have considered as mainly involved in the development of attachment are: genetics, neurobiology and cognitive functioning. Through internal processes involving these issues, information from the dyadic interaction environment generates in the infant predictions and representations of the Self, of the world, and of the caregivers. We analyzed the development of the attachment system using the period spent in the intrauterine environment as the temporal origin. The prenatal phase, in fact, is the period during which an individual is exposed to the most massive dose of organic information from what in many cases is the primary caregiver: the mother.

What emerges from our work is that genetics plays a relevant role in attachment, first of all because it conveys innate instincts that induce the formation of such a bond. Its role, however, is not sufficient to explain the qualitative development of attachment, and no specific “attachment gene” or “attachment genetic pathway” has been identified to date. Evidence derived from studies of candidate genes brings light to the presence of genes that, rather, serve as risk or protective factors for insecure attachment or as susceptibility factors that can enhance the positive or negative effects of environmental contingencies on various dimensions of attachment, particularly security. Genes such as COMT, FBKP5 and 5HTTLPR are implicated in stress response, and specific polymorphisms of these genes have been shown to contribute to attachment security regardless of the quality of parental care (Craig et al., 2021; Rasmussen and Storebø, 2021). An interesting process involving attachment and genetics is the epigenetic process. It provides insight into how the quality of early caregiving experience accesses the genetic code, influencing the organism at a molecular level. One example is the NR3C1 gene which appears frequently methylated in children with poorly supportive attachment experience and high levels of attachment anxiety (Murgatroyd et al., 2015). An additional aspect of interest is the transmissibility of epigenetic modifications from parent to child (Fujiwara et al., 2019), which could contribute to the explanation of how the attachment style of parents influences that of their children.

The neurobiology perspective discusses the neurobiological mechanisms involved in attachment dynamics and their relationship with pre-natal and post-natal experience. The attachment system is a means of maintaining physical and emotional security, and its activation is triggered by disturbances to psychophysical balance. The caregiver’s role is to co-regulate the infant’s internal state, minimizing the energy the infant must expend in restoring homeostasis. Attachment style reflects the capacity for self- or co-regulation of internal states, thus the modulation of the underlying neural and physiological activation. We assume the prenatal period to be of considerable importance, as social learning processes are already underway through sensory and organic stimuli, laying the foundation for subsequent general-domain neural and physiological memories. Of particular interest for this section is the NAMA model proposed by Long et al. (2020), which aims to explain the neural mechanisms of regulatory processes in secure and insecure organized attachment patterns. The authors reviewed the attachment neurofunctional pathways indicating the presence of four modules involved in attachment processes and illustrated how secure, insecure avoidant, and insecure ambivalent attachment differ in their response strategies to stressful events. Attachment security coincides with an increase in the activity of neural and neurotransmitters’ pathways of the (self-) regulation module and a decrease in the aversion module. Insecure attachment patterns are marked by the difficulty of restoring the homeostatic state and experiencing the derivative sensation of security. Empathy, theory of mind, social perception, and social learning are the neural and behavioral processes associated with the second-person perspective. Attachment style can impact these processes, with securely attached individuals demonstrating higher levels of these skills compared to insecurely attached individuals. Since there is no “attachment specific neural pathway,” the quality of attachment experiences is thought to be crucial for general-domain functions, such as biobehavioral, cognitive, and psychosocial abilities.

From the cognitive functioning perspective, it appears plausible that a child in the first year of life is capable of constructing mental representations. The formation of IWM in infants is linked to the formation of cognitive capacities such as the recognition and discrimination of persons, their permanence, the memory of past experiences, the creation of expectations based on experience, and the understanding of the meaning of caregiver’s behaviors and emotions. Infants as young as the first month of life can recognize and discriminate between faces, and by 4 months of age, they can retain memories of social events that generated a stress response for 15 days. By the third month of life, the maturation of implicit memory is completed, and there is a progressive complexification of motor sequences that can demonstrate its existence. Infants build different expectations based on past experiences with specific adults and can predict future caregivers’ behaviors. Social expectations could be innate, and infants as young as 9 months have demonstrated expectancies on positive social behaviors while observing conflict in an interactive group of adults (Sherman et al., 2015). It’s even hypothesized that such representations may start forming prior to birth. However, mental representations alone may not fully explain the intricate dynamics that shape an individual’s organization, including early experiences, child-specific traits, and environmental events. Embodied cognition is a field of cognitive science that emphasizes the importance of the body and environment in cognitive abilities. It suggests that mental processes are not solely computational, and the brain is not the only seat of cognition. Crossing the neurobiology perspective, the embodied approach could shed light on how sensory and metabolic information imbues certain multi-domain acquisitions with meaning (Petters and Waters, 2014; Beckes et al., 2015; Atzil et al., 2018). We propose that attachment and its embodied patterns could start evolving during pre-natal period, influenced by the neurochemical and sensory input the fetus and then the infant receives.

During prenatal development, the fetus is connected to the mother’s experiences and mental states through various stimuli and hormonal exchanges. This interaction, forming what could be termed an embodied dyad, involves adaptive response to maternal states through homeostatic and epigenetic organization, suggesting that dyadic exchanges which shape attachment patterns may begin before birth. This information highlights the importance of considering prenatal development when exploring human attachment.

The genetic heritage of the fetus during prenatal phase contains trans-generational information that are influenced by the social environment through epigenetic processes. The mother’s physical and mental health during pregnancy greatly impacts the fetus’s programming and creates what we could refer to as molecular memory. In other words, the social environment can affect the way genes are expressed and can impact the development of the fetus.

Beginning with genetic instructions, the fetus organism needs to learn from environmental statistics to put in place homeostatic mechanisms for its own survival. This is why homeostasis can be considered a memory system first formed in the intrauterine environment dependent on the mother’s psychophysical state. During late gestation, the fetus develops sensory units as well as a cortical structure sufficiently evolved to enable it to register sensory stimuli and retain them in memory until the postnatal period to guide adaptive behaviors. Sensory memory is thus an additional component that guides attachment to specific caregivers and particularly to the biological mother, generating increased responsiveness to events related to her in the immediate postnatal period.

At birth, social stimuli are associated with internally produced sensations and their valence. For example, feeding on the maternal breast can create positive memories and a sense of security through specific neurofunctional pathways. Emotional memory is a basic system that relies on subcortical centers and may develop as early as the prenatal period. Emotional memories are activated during stress and help regulate response to discomfort. Cognitive representations that allow predicting caregiver behavior develop later and are closely linked to emotional memories. The attachment system, thus, represents a multifaceted framework of social and emotional learning that starts in the womb and continues to evolve and mature through the early years of life, with different components developing at varying stages.

We conclude that studies from various bottom-up perspectives converge to demonstrate the need for a neuro-cognitive model of attachment that regulates them according to top-down principles. The Active Inference Theory, proposed by Friston in 2006, fits well as an overarching model of attachment, integrating bottom-up perspectives. This theory originates from neuroscience and is rooted in pure sciences such as mathematics and physics. It conceptualizes the brain as a predictive machine that generates forecasts based on the model of the world it constructs. Furthermore, this theory integrates the Free Energy Principle, according to which all living organisms persist by minimizing the dissipation of energy or, in other terms, are constantly engaged in reducing discrepancies between what the brain predicts and what happens around it (Parr et al., 2022). This mechanism is not to be understood only as a cognitive or neurobiological mechanism, but as something that involves the entire organism. The Free Energy Principle and the conception of the brain as a predictive machine, central in Active Inference Theory, provide an ideal framework to unify research on the development of the attachment system from various fields. Using this framework, it is possible to harmonize attachment studies under a single theory, where neurobiological and psychological events and subjectivity are regulated by the same principles. This approach allows explaining how the integration between innate predisposition and the quality of care received in the early years of life cooperate in the formation of predictive embodied models of the world that influence behaviors and neurobiological response strategies throughout life. Additionally, the applicability of these principles to organisms of various complexity would enhance the analysis and understanding of the continuum of attachment development from the prenatal to the postnatal period.

Future studies could further explore the integration of attachment studies under the Active Inference framework, offering new perspectives and insights into how early experiences influence cognitive and relational development. Moreover, adopting an Active Inference framework could not only enrich the understanding of the perspectives explored in our study on attachment (neurobiological, genetic, and cognitive perspective) but also extend to many others. Furthermore, it would allow considering not only the child’s system itself but also the broader interaction system, such as the dyad (mother–child), the triad (mother–father-child), and so on. This neat interdisciplinary approach could offer a more complex and detailed view of attachment dynamics, enriching our understanding of the origins psychological, neurobiological, and behavioral outcomes.

## Author contributions

ES: Writing – original draft, Methodology, Investigation, Conceptualization. MB: Writing – review & editing, Visualization, Resources, Methodology, Investigation, Conceptualization.
